# Effects of Corni fructus on ovalbumin-induced airway inflammation and airway hyper-responsiveness in a mouse model of allergic asthma

**DOI:** 10.1186/1476-9255-9-9

**Published:** 2012-03-23

**Authors:** Seung-Hyung Kim, Bok-Kyu Kim, Young-Cheol Lee

**Affiliations:** 1 Institute of Traditional Medicine & Bioscience, Daejeon University, Daejeon 300-716, Republic of Korea; 2 Department of Herbology, College of Oriental Medicine, Sangji University, Wonju 220-702, Republic of Korea

**Keywords:** Corni fructus, Asthma, Eosinophil, IL-5, CCR3

## Abstract

**Background:**

Allergic asthma is a chronic inflammatory lung disease that is characterized by airway hyperresponsiveness (AHR) to allergens, airway oedema, increased mucus secretion, excess production of T helper-2 (Th2) cytokines, and eosinophil accumulation in the lungs. Corni fructus (CF) is a fruit of *Cornus officinalis *Sieb. Et. Zucc. (Cornaceae) and has been used in traditional Korean medicine as an anti-inflammatory, analgesic, and diuretic agent. To investigate the anti-asthmatic effects of CF and their underlying mechanism, we examined the influence of CF on the development of pulmonary eosinophilic inflammation and airway hyperresponsiveness in a mouse model of allergic asthma.

**Methods:**

In this study, BALB/c mice were systemically sensitized to ovalbumin (OVA) by intraperitoneal (i.p.), intratracheal (i.t.) injections and intranasal (i.n.) inhalation of OVA. We investigated the effect of CF on airway hyperresponsiveness, pulmonary eosinophilic infiltration, various immune cell phenotypes, Th2 cytokine production, and OVA-specific immunoglobulin E (IgE) production.

**Results:**

The CF-treated groups showed suppressed eosinophil infiltration, allergic airway inflammation, and AHR via reduced production of interleuin (IL) -5, IL-13, and OVA-specific IgE.

**Conclusions:**

Our data suggest that the therapeutic effects of CF in asthma are mediated by reduced production of Th2 cytokines (IL-5), eotaxin, and OVA-specific IgE and reduced eosinophil infiltration.

## Background

Allergic asthma generally presents with symptoms of wheezing, coughing, breathlessness, and airway inflammation. It is a chronic inflammatory disease of the airways, characterized by airway eosinophilia and goblet cell hyperplasia with mucus hypersecretion to inhaled allergens and nonspecific stimuli [1,2]. In particular, eosinophilic inflammation is considered the hallmark of airway inflammation in asthma [3]. The inflammatory process in allergic asthma is dominated by Th2 cells that produce IL-4, IL-5, and IL-13 [4], which activate eosinophils and induce the production of IgE by B cells [5,6].

CF is a Korean traditional medicinal herb with tonic, analgesic, and diuretic activity and has been commonly used to facilitate liver and kidney function, reduce urination, and decrease perspiration. We previously reported that CF has anti-allergic activity in mouse splenic B cells and IC-2 mast cells [7]. Sung *et al. *[8] reported that aqueous extracts of CF have anti-inflammatory and analgesic activities in murine RAW 264.7 macrophage cells. Moreover, Du *et al. *[9] reported that the polysaccharides in crude and processed CF enhance nonspecific immunity, specific humoral immunity, and specific cellular immunity in immunosuppressed mice, and processing of CF with wine markedly increases the activity of the polysaccharides. Other biological activities of CF include hypoglycaemic, anti-neoplastic, and anti-microbial effects, as well as the improvement of liver and kidney function [10-12]. However, no study thus far has addressed the anti-asthmatic and anti-inflammatory activity of CF *in vivo*.

The aim of this study was to evaluate the effect of CF extract on Th1 and Th2 cytokines as well as the development of allergic airway inflammation and airway hyperresponsiveness in a mouse model of allergic asthma.

## Methods

### Plant material and preparation of extracts

CF samples were purchased from Wonju Jaeil Korean Herbs Co. Ltd. (Wonju, Korea) in May 2011 and identified by Professor Young-Cheol Lee of the College of Oriental Medicine at Sangji University in Wonju, Korea, and a voucher specimen (CF) was deposited in our laboratory (Department of Herbology, College of Oriental Medicine, Sangji University Wonju 220-702, Republic of Korea). The plant material (200 g) was extracted 3 times with H_2_O. The extract was then filtered and evaporated on a rotatory evaporator (Rotary evaporator, BUCHI B-480, Switzerland) and finally dried using a freeze drier (Freeze dryer, EYELA FDU-540, Japan) to yield the extract CF (27.1 g). The yield (w/w) of the extract was approximately 13.5%.

### Animals

Five-week-old female BALB/c mice were obtained from Daehan Biolink Co. LTD. (Eumsung, Republic of Korea). Our study was approved by the committee for animal welfare at Daejeon University. Moreover, all animal procedures were conducted in accordance with the guidelines of the Institutional Animal Care and Use Committee of the Korea Research Institute of Bioscience and Biotechnology (Daejeon, Republic of Korea).

### OVA sensitization and inhalation

**Figure 1:**
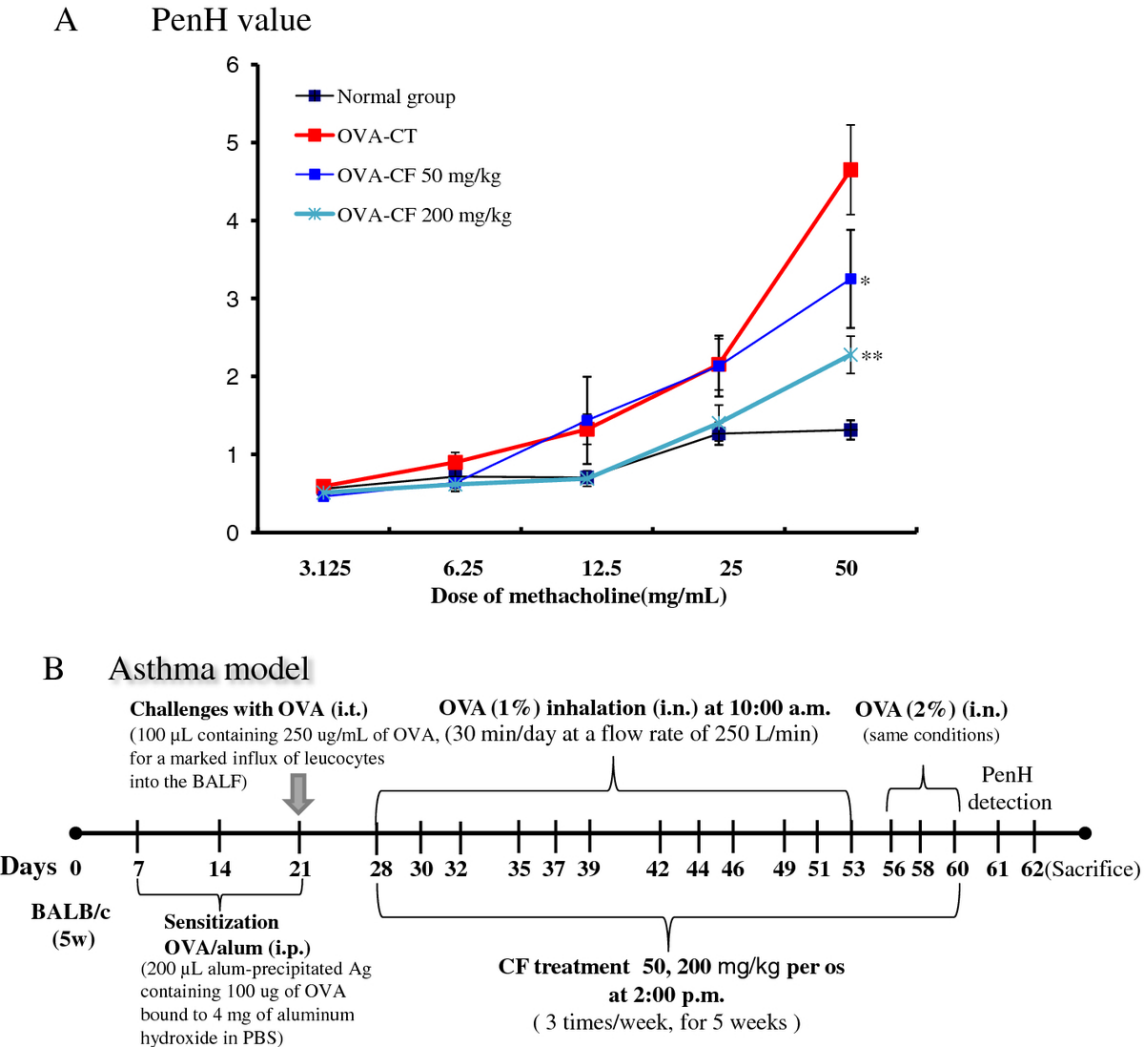
**Effect of CF on airway hyperresponsiveness in OVA-challenged mice**. Mice were sensitized and challenged by OVA as described in the Materials and methods. The airway responsiveness to aerosolized methacholine was measured with a Buxco box, as described in the Materials and methods. The mice were placed in the main chamber and were nebulized first with PBS and then with increasing doses (3.125 to 50 mg/mL) of methacholine for 3 min for each nebulisation (A). Schematic representation showing the protocol to induce asthma model (B). The data represent the mean ± SEM from 5 independent experiments. *P < 0.05, **P < 0.01 for the control goup versus the CF-treated groups.

Based on a modification of a previously described protocol [13], OVA (500 μg/mL) in PBS was mixed with equal volumes of 10% (w/v) aluminum potassium sulphate (alum; Sigma) in distilled water, incubated for 60 min at RT after pH adjustment to 6.5 by using 10 N NaOH, and centrifuged at 750 × *g *for 5 min. The OVA/alum pellet was resuspended to the original volume in distilled water. As shown in Figure 1B, all mice were immunized on 3 different days (i.e., 7, 14, or 21 days) by i.p. injections of 0.2 mL alum-precipitated antigen containing 100 μg of OVA (Sigma-Aldrich Korea, Korea) bound to 4 mg of aluminum hydroxide (Sigma-Aldrich Korea, Korea) in PBS. Seven days after the challenges via i.t. injections of 100 μL (250 μg/mL) of OVA (on day 21) in the back of the tongue for a marked influx of leucocytes into the BALF, the mice were exposed to aerosolized OVA for 30 min/day, 3 days/week for 5 weeks (at a flow rate of 250 L/min; 1% OVA in normal saline for the first 4 weeks and 2% OVA in normal saline for the last week). CF (50 and 200 mg/kg, Sigma-Aldrich Korea, Korea) was orally administered 3 times a week for the last 5 weeks. One day after the last OVA exposure (2% OVA inhalation), the airway hyperresponsiveness was determined, and bronchoalveolar lavage fluid (BALF), lung cells, and serum samples were collected for further molecular analyses.

### BALF

Immediately following the assessment of AHR, the mice were killed with an i.p. injection of sodium pentobarbitone (100 mg/kg). The trachea was cannulated, and BALF was obtained by washing the airway lumen. Briefly, cells of the lungs were recovered by flushing 1 mL of BALF (1 mM EDTA, 10% FBS, PBS) into the lungs via the trachea. Total cell counts were determined after 100 μL of fluid was cytospun onto glass slides using a Cytospin centrifuge (Cellspin, Hanil,, Korea) (400 × *g *for 4 min). Differential cell counts were performed after staining with a Diff-Quik Stain Set (Baxter Healthcare Corp., Miami, Florida, USA). The supernatant of the BALF was stored at -25°C for the determination of cytokine levels.

### Digestion of pulmonary tissue and cell preparation

Single-cell suspensions from lung tissues and BALF were isolated by mechanical disruption in RPMI 1640 medium supplemented with 2 mM L-glutamine, 100 U/mL penicillin, 100 μg/mL streptomycin, 50 μM 2-mercaptoethanol, 20 mM HEPES, and 2% heat-inactivated foetal bovine serum (FBS, GIBCO, Grand Island, NY). Briefly, the lungs were removed from the thoracic cavity. After mincing using sterile scalpels, the tissue was incubated in PBS containing 1 mg/mL collagenase IV and 2 mg/mL dispase for 40 min at 37°C in a sterile polypropylene tube. After incubation, the lung tissue was vigorously pipetted up and down to further dissolve the remaining tissue clumps and then filtered using a 70-μm cell strainer (Falcon, Le Pont de Claix, France). The total number of cells was counted manually using a haemocytometer chamber (Fisher), and 2-4 × 10^3 ^cells were spun onto glass slides (Cytospin centrifuge, Cellspin, Hanil, Korea) (400 × *g *for 4 min). Differential counting was performed according to standard morphologic criteria.

### Determination of AHR

AHR in the mice was estimated using a previously described method with modifications [13]. A Buxco system (Biosystem XA; Buxco Electronics Inc, Troy, Conn) was used to evaluate the extent of airway constriction in different groups of mice according to the protocol described previously.

The Penh value is equal to Pause × PEF/PIF, where Pause = (Te-Tr)/Tr (PIF, peak inspiratory flow; PEF: peak expiratory flow; Te, expiratory time; Tr, relaxation time). In this experiment, the mice were aerosolized with OVA for 30 min/day, 3 days/week for 5 weeks. At 24 h after the final inhalation, the mice were given aerosolized normal saline, followed serially by 3.125, 6.25, 12.5, 25, and 50 mg/mL methacholine (Sigma). The airway reactivity was then monitored for 30 min. Differences in the Penh value between the groups were evaluated using Mann-Whitney test.

### Haematoxylin-eosin (H&E), masson-trichrome (M-T), periodic acid-Schiff (PAS) staining and BALF cytospin

The BALB/c mice were treated with OVA for 5 weeks (3 times a week) to induce asthma. Two experimental groups were treated with different concentrations of CF for the last 5 weeks (5 times/week). At the end of the experiment, the lungs were removed and analyzed histologically using a modification of a previously described protocol [13].

Briefly, the lung tissue was embedded in paraffin, cut into 3-μm-thick sections, and stained with H&E or M-T solution. The tissue was subsequently mounted and coverslipped with Dako mounting medium (Dakocytomation; Carpinteria, CA). The degree of inflammatory cell infiltration in the airway was scored in a double-blind manner by 2 independent observers. The degree of peribronchiolar and perivascular inflammation was evaluated on a subjective scale of 0-2, as modified from a previously described protocol [13]. Periodic acid-Schiff (PAS) staining was performed to identify mucus secretion in the lung tissue. Frozen sections (30-mm thick) were generated for each tissue. The sections were mounted on gelatin-coated slides, stained with PAS reagents, dehydrated, and coverslipped with permount. The PAS-positive goblet cells were counted manually, normalized against the length of the bronchial epithelial perimeter on the basal side, and expressed as the number of PAS-positive cells per millimetre of basement membrane.

### Antibodies and flow cytometric analysis

All antibodies (anti-CD3, CCR3, CD11b, Gr-1) for flow cytometric analysis were purchased from Becton Dickinson (BD) PharMingen (San Diego, CA). Cells from lung tissues and BALF were stained with the indicated antibodies in a staining buffer (PBS containing 1% FBS and 0.01% NaN3) for 10 min on ice, and analyzed by 2-colour flow cytometry on a FACSCalibur using CellQuest software (BD Biosciences, Mountain View, CA).

### Enzyme-linked immunosorbent assay (ELISA)

Interleukin (IL-4, IL-5, and IL-13) and IFN-γ production in the BALF and the presence of anti-OVA IgE in the serum of the mice (n = 5) were analysed by ELISA according to the manufacturer's instructions with a monoclonal antibody-based mouse interleukin ELISA kit (R&D system). For OVA-specific IL-4 and IFN-γ production, spleen cells were suspended in RPMI 1640 medium supplemented with 2 mM L-glutamine and 5% foetal bovine serum and then cultured for 48 h at a density of 1 × 10^5 ^cells/well in 96-well culture plates (Corning Inc, Cambridge, Mass) with or without 1 μg/mL of OVA in a humidified atmosphere of 5% CO_2 _in air at 37°C. The culture supernatants were collected and assayed for IFN-γ and IL-4 induced by OVA by using ELISA. All data represent the mean and standard deviation of at least 3 separate replications and were compared using analysis of variance (ANOVA).

### Fingerprint high performance liquid chromatography (HPLC) analysis

#### Chemicals and reagents

Morroniside was purchased from NPC BioTechnology (Yeongi, Korea), and loganin and ursolic acid were purchased from Sigma (USA). The purity of each compound was determined to be above 98% by HPLC analysis. The HPLC-grade reagents acetonitrile and water were obtained from J.T. Baker (Phillipsburg, NJ, USA). The other chemicals were of analytical grade.

#### Chromatographic system and conditions

The analysis was performed using a Waters HPLC system equipped with a Waters 600 pump, a Waters 996 PDA detector, an Empower system controller, and an HPLC column (Optimapak C18 column, 4.6 × 250 mm, i.d., 5-μm particle size). The mobile phase for the HPLC analysis consisted of water and acetonitrile (86:14 for morroniside, loganin, and CF analyses; 10:90 for ursolic acid and CF analysis). The column temperature was maintained at 40°C. The analysis was performed using a flow rate of 1.0 mL/min with PDA detection (240 nm for morroniside and loganin analysis, 200 nm for ursolic acid analysis). The injection volume was 20 μL.

### Statistical analysis

The data were analyzed using a one-way ANOVA to determine statistically significant variance between the groups for each end point assessed. Statistical significance between groups was then calculated by using the nonparametric Mann-Whitney test followed by Dunnett's multiple comparison test (IBM SPSS statistics version 19.0 statistic software, Inc, IBM, USA). The difference between the normal group and the control group (OVA + vehicle) was obvious; for this reason, the statistical significance between the normal group and the control group is not shown in the figures and tables. The results (presented as mean ± standard error of mean) were defined as statistically significant for the following P values: < 0.05 (*), < 0.01 (**), or < 0.001 (***).

## Results

### Inhibitory effect of CF on AHR

In a mouse model of allergic asthma, we evaluated the effects of CF delivered by nebulisation only or in combination with oral administration. Both CF treatments were equally efficient for reducing AHR to methacholine, as determined by whole-body plethysmography (Figure 1A).

The Penh value was measured using a Buxco system on day 1 after the final inhalation, and the samples were immediately collected. Methacholine treatment is useful to determine the distinct effect of drugs on the Penh value through induction of AHR. Compared with normal mice, in OVA-sensitized mice, the dose-response curve of the Penh value was shifted to the left (Figure 1A). As shown in Figure 1A, relative to animals sensitized with OVA (control group), the AHR to methacholine was reduced in the CF-treated (50, 200 mg/kg) mice (P < 0.01, P < 0.05).

### Histological analysis of lung sections

**Figure 2:**
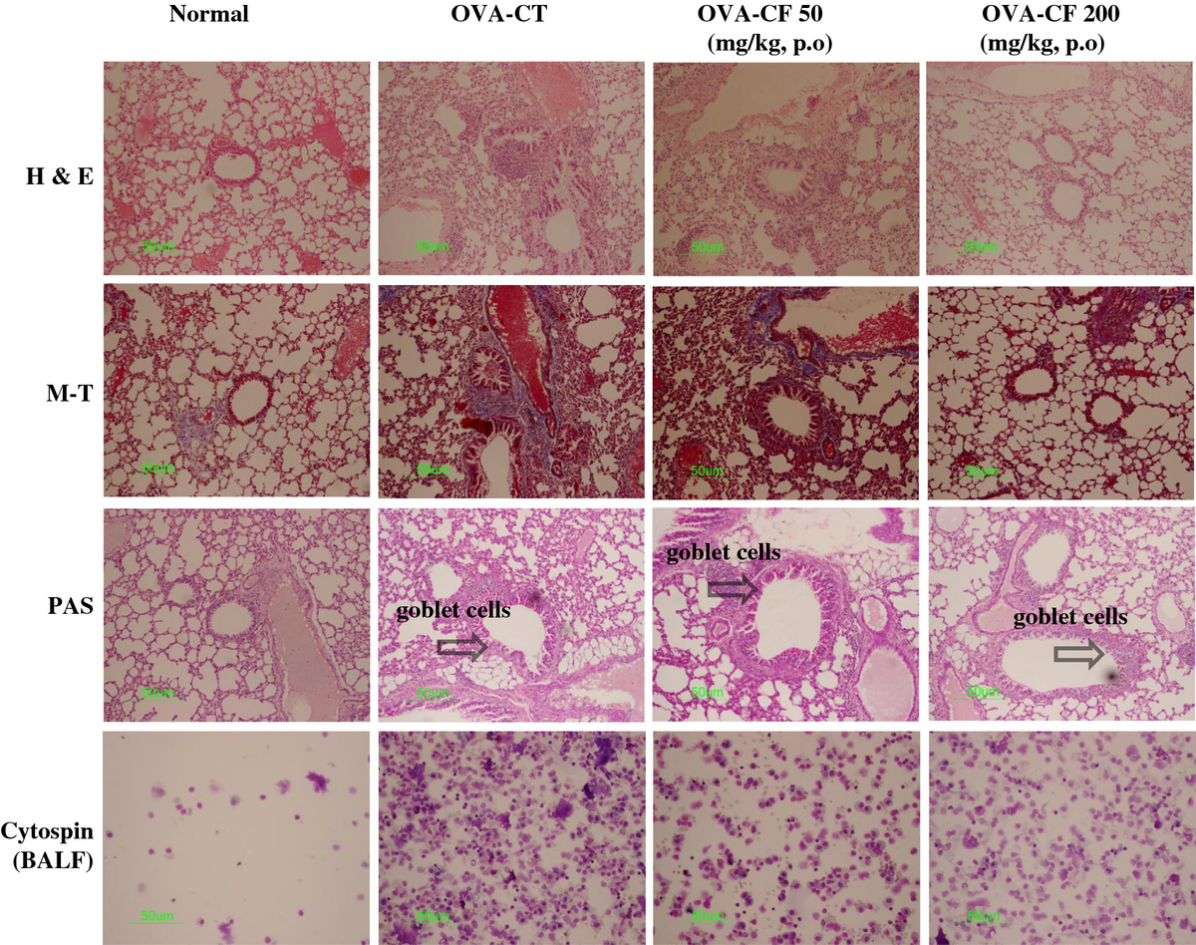
**Effect of CF on airway inflammation (H&E, M-T, and PAS staining) in lung tissue and BALF of OVA-induced asthmatic mice**. At the end of the experiment, the lungs were fixed, and a histologic analysis was performed. Lung sections were obtained for normal and asthmatic mice treated with vehicle (control), CF (50 mg/kg), and CF (200 mg/kg). H&E: hematoxylin-eosin stain, M-T: Masson trichrome stain, PAS: periodic acid-Schiff Stain, N: Normal BALB/c mice, CT (control): Ovalbumin inhalation + vehicle, OVA + CF (50, 200 mg/kg).

As shown in Figure 2, the inflammation in the peribronchial and perivascular regions of mice receiving CF significantly decreased compared with that in mice in the OVA group. The OVA-challenged mice and CF-treated mice showed inflammatory histological changes when compared with saline-challenged normal mice. We also found infiltration of leukocytes in sections of the lungs from OVA-challenged control mice as well as a distinct inflammatory infiltration and erosion in the peribronchial and perivascular areas. The peribronchial and perivascular inflammatory infiltrate consisted of eosinophils and mast cells that were mixed with lymphocytes. Eosinophil infiltration was mainly observed in the peribronchial regions of the lung. In contrast, histological sections from CF-treated mice indicated reduced airway inflammation in the lung tissue (Figure 2).

To investigate the presence of mucus in the airway, PAS-positive and PAS-negative epithelial cells were analyzed in individual bronchioles. Goblet cell hyperplasia and mucus hyperproduction were evaluated by means of PAS staining and quantification of PAS-stained cells. The OVA-challenged control mice showed significantly increased numbers of PAS-positive cells compared with saline-challenged normal mice; moreover, a greater reduction in the mean number of PAS-stained goblet cells was observed in the CF-treated (50, 200 mg/kg) asthma mice than in the OVA-sensitized/challenged mice (Figure 2).

### Inhibitory effect of CF on airway eosinophil accumulation and influx of inflammatory cells into the lungs and BALF

In total, 2.19 ± 0.39 × 10^7 ^cells were obtained from lungs of the saline-challenged group; few eosinophils were detected in this group. In contrast, the total number of lung cells (5.58 ± 0.3 × 10^7^) and eosinophils in the BALF cytospin of the OVA-challenged mice was significantly higher than that in the saline-challenged group.

**Figure 3:**
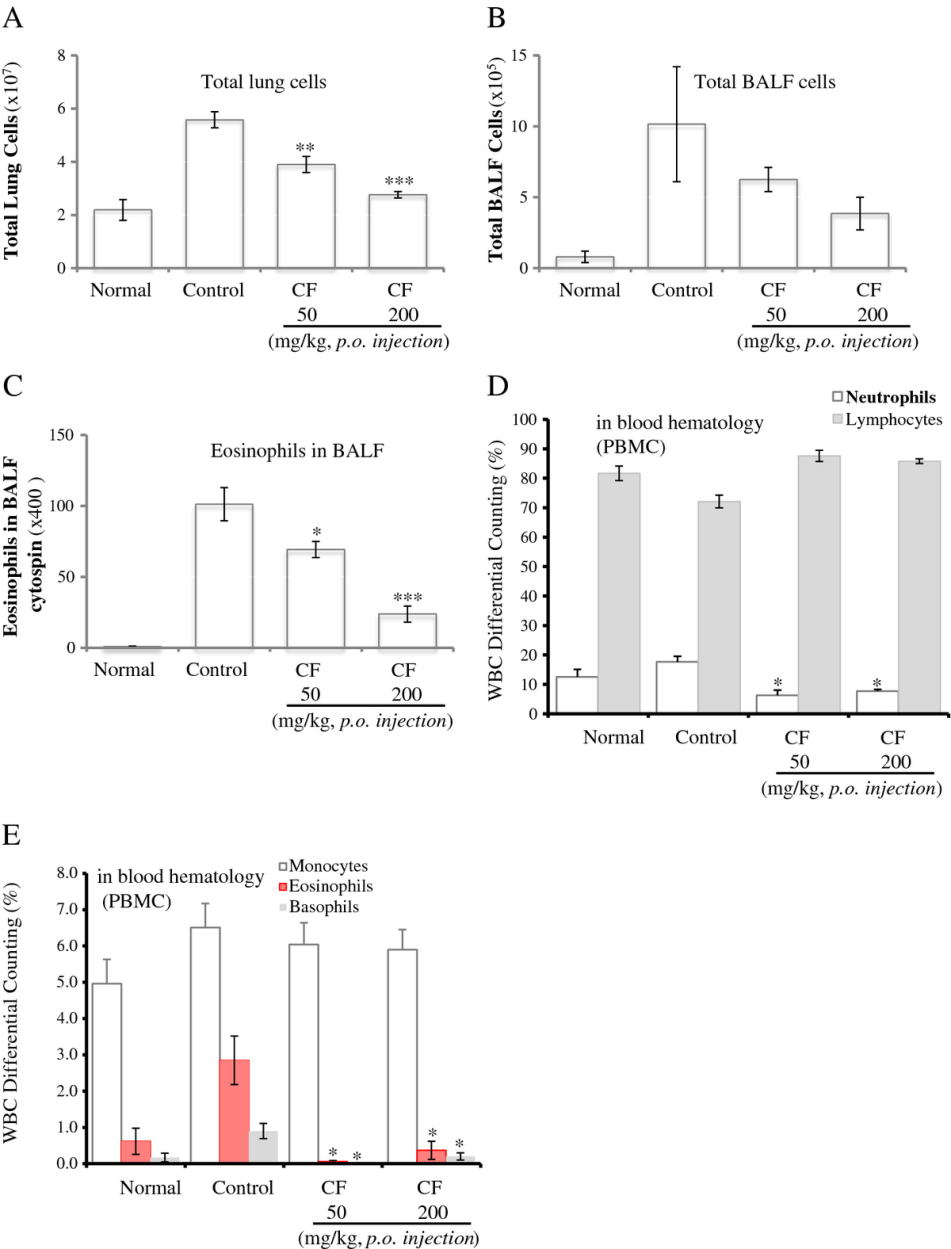
**Effects of CF on total lung cells, total BALF cells, eosinophils in the BALF, and PBMCs**. As described in the Materials and methods, the lung and BALF were harvested 24 h after the last OVA challenge. The total inflammatory cell numbers in the lung and BALF were counted, and cell classification was performed on a minimum of 200 cells to classify lymphocytes. The results are expressed as the mean ± SEM (n = 5). The statistical significance of differences between control and treatment groups was assessed by ANOVA or the nonparametric Mann-Whitney test followed by Dunnett's multiple comparison test (*P < 0.05, **P < 0.01, ***P < 0.001). N: Normal BALB/c mice, CT: Ovalbumin inhalation + vehicle, OVA + CF (50, 200 mg/kg).

The total number of lung cells was significantly reduced in CF-treated (50, 200 mg/kg) mice compared with control mice; moreover, CF treatment (50, 200 mg/kg) decreased the absolute number of eosinophils in the BALF as well as neutrophils and eosinophils in the PBMC (Figure 3C, D, E).

### Inhibitory effect of CF on the absolute number of immune cell sub-types in OVA-induced asthmatic lungs and BALF

**Figure 4:**
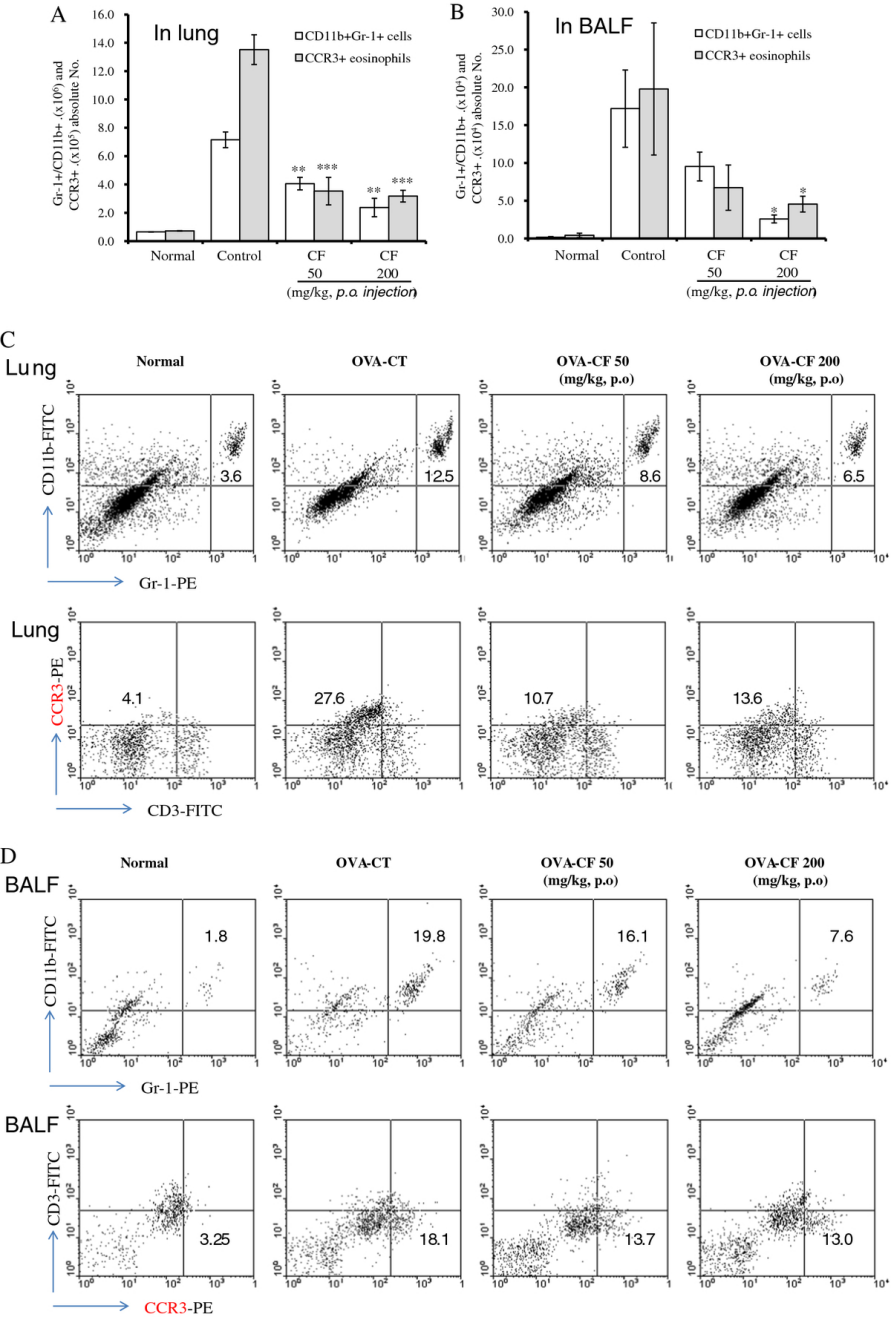
**FACS analysis of various immune cell sub-types in the lung and BALF**. The absolute numbers of various immune cell subtypes in the lung were counted (A, B) (described in Materials and methods). The lung and BALF cells were stained with FITC-conjugated mAb to CD3 and CD11b as well as PE-conjugated mAb to Gr-1 and CCR3. A dot-plot pattern is shown for the representative flow cytometric profile for the gated CD3-/CCR3+ and Gr-1+/CD11b + cells (C, D). The results are expressed as the mean ± SEM (n = 5). The statistical significance of differences between the control and treatment groups was assessed by ANOVA or the nonparametric Mann-Whitney test followed by Dunnett's multiple comparison test (*P < 0.05, **P < 0.01, ***P < 0.001). N: Normal BALB/c mice, CT: Ovalbumin inhalation + vehicle, OVA + CF (50, 200 mg/kg).

Flow cytometric analysis was used to evaluate the effect of CF on immune cell sub-types. The number of CD3-, CCR3-, CD11b-, and Gr-1-positive cells in the lungs and BALF of OVA-challenged mice was higher than that in the saline-treated group, and generally, the number in CF-treated mice was significantly lower than that in the OVA-challenged mice (Figure 4).

The effects of CF on leukocyte subsets in the lungs and BALF of OVA-treated mice, compared with the control group, included changes in the number of Gr-1^+^/CD11b^+ ^granulocytes as well as CD3-/CCR3+ eosinophils; further, the decreases in CD3-/CCR3+ eosinophils were accompanied by concurrent decreases in eosinophils in the BALF (Figure 3C).

### Inhibition of Th2 cytokines (*in vivo and in vitro*) and OVA-specific IgE production in BALF and serum

To determine whether CF influenced Th2 cytokine secretion in the BALF, the levels of IL-4, IL-5, and IL-13 were measured using ELISA after the final challenge.

**Figure 5:**
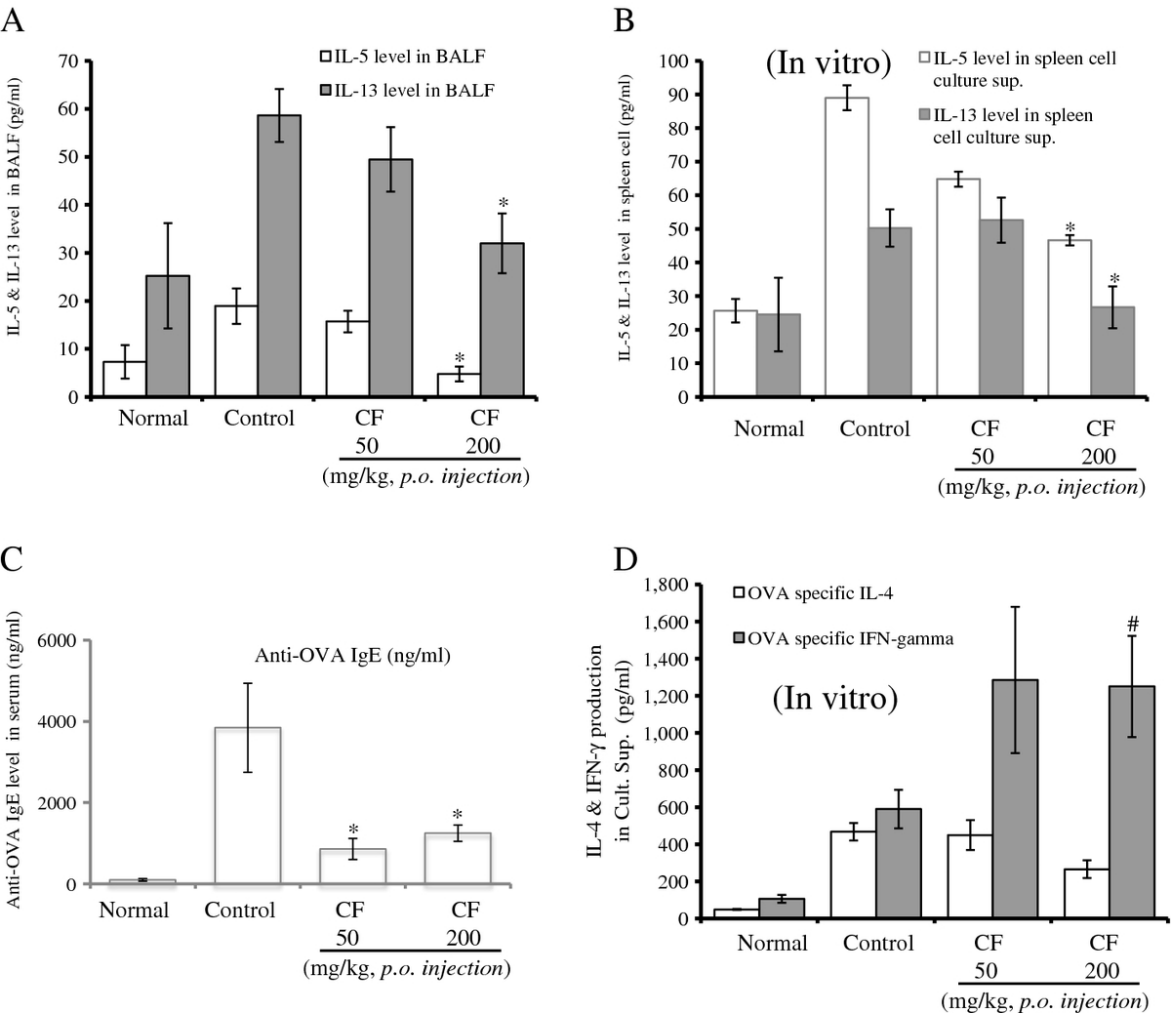
**Effect of CF on Th2 cytokines (IL-5, IL-13) in the BALF and OVA-specific IgE in the serum, and immunomodulatory effects of CF on OVA-specific Th1/Th2 cytokines produced by spleen cells (described in Materials and methods)**. The results are expressed as the mean ± SEM (n = 5). The statistical significance of differences between the control and treatment groups was assessed by ANOVA or the nonparametric Mann-Whitney test followed by Dunnett's multiple comparison test (*P < 0.05, **P < 0.01, ***P < 0.001). N: Normal BALB/c mice, CT: Ovalbumin inhalation + vehicle, OVA + CF (50, 200 mg/kg).

As shown in Figure 5A and 5B, the IL-5 and IL-13 levels were significantly reduced in CF-treated (200 mg/kg) mice and in the spleen cell-culture supernatant. An important component of the allergic asthma model is the production of OVA-specific IgE. Therefore, the levels of anti-OVA IgE were measured in serum from the OVA-challenged mice as well as the mice treated with PBS and CF. OVA-specific IgE levels in the serum from OVA-induced asthmatic mice were significantly higher than those in the serum from the normal mice (PBS only). Further, the CF-treated mice had significantly reduced levels of OVA-specific IgE (Figure 5C). We also measured IL-4 and IFN-γ in the culture supernatants by ELISA and found that CF (200 mg/kg) inhibited Th2 cytokine (IL-4) production and increased Th1 cytokine (IFN-γ) levels in splenocytes (Figure 5D).

### HPLC analysis of morroniside, loganin, and ursolic acid from CF-treated mice

**Figure 6:**
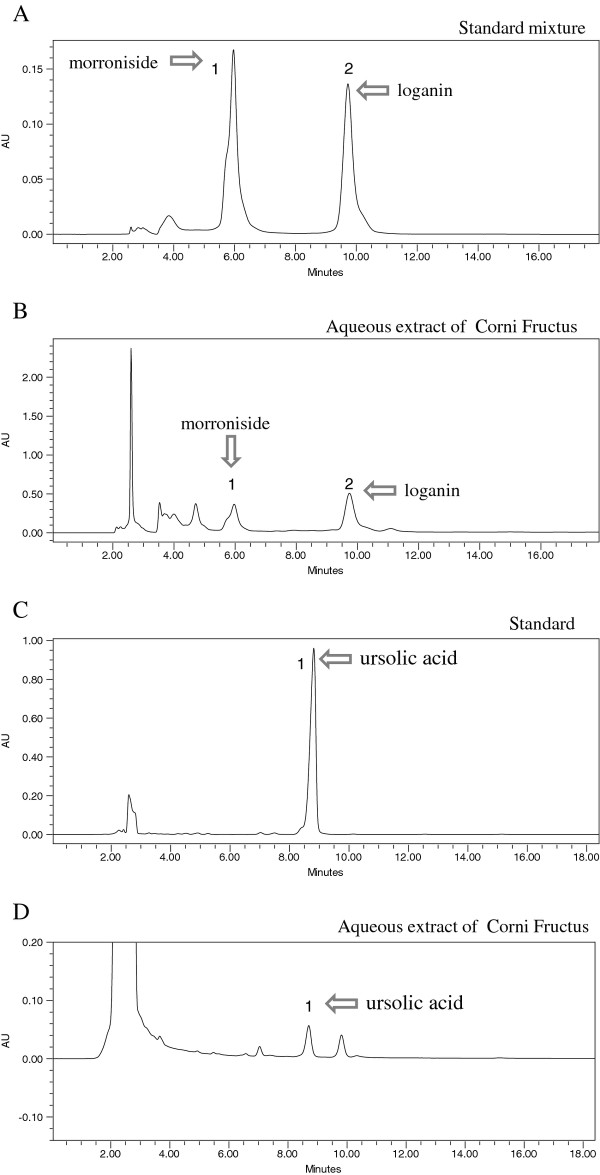
**HPLC chromatogram of standard mixture (A) and *C. officinalis *(B) at 240 nm**. Morroniside (1), loganin (2). *C. officinalis *and 2 standards were subjected to HPLC analysis. An Optimpak C18 (4.6 × 250 mm) column was eluted with water and acetonitrile (86:14) at flow rate of 1.0 mL/min. The HPLC chromatogram is shown for the ursolic acid standard (C) and *C. officinalis *(D) at 200 nm. *C. officinalis *and ursolic acid were subjected to HPLC analysis. The Optimpak C18 (4.6 × 250 mm) column was eluted with water and acetonitrile (10:90) at flow rate of 1.0 mL/min.

The retention times of morroniside, loganin, and ursolic acid were approximately 5.9 min, 9.9 min, and 8.3 min, respectively. Figure 6 shows the chromatograms of reference compounds and the *C. officinalis *extract; the eluent was detected at 240 nm. The peaks of the 3 major components were identified as morroniside, loganin, and ursolic acid.

## Discussion

In allergic asthma, the activation of allergen-induced Th2 cells in the lungs leads to eosinophilic lung inflammation, increased secretion of mucus, and recurring bronchospasm. Eventually, with repeated allergen exposure, the bronchial smooth muscle and basement membrane thicken, lung tissue is destroyed, and lung function is impaired [14]. Activated T lymphocytes in the airways secrete the Th2 cytokine IL-5, which regulates eosinophilic inflammation by regulating the proliferation, differentiation, and activation of eosinophils [15,16].

Previous studies have shown that Corni fructus can control airway allergy and inflammation [17] and that its main components, ursolic acid and oleanolic acid, have anti-inflammatory and anti-oxidative effects [18-20]. Ursolic acid influences basal airway mucin release [21], and ursolic acid and oleanolic acid have been reported to affect adenosine triphosphate (ATP)-induced airway mucin release from epithelial cells of the tracheal surface of cultured hamster [22]. Other studies have showed that CF has anti-diabetic activity in rats with streptozotocin-induced diabetes [23,24]. More than 150 compounds have been isolated from CF [25], and some components (e.g., ursolic acid and oleanic acid) have shown anti-diabetic effects [26,27]. However, the exact mechanisms underlying the biological efficacy of CF remain unclear, and its influence has not been studied thus far in an asthma model.

The recruitment of eosinophils to the airways is a characteristic of asthma, and the degree of eosinophilia is correlated with the severity of the disease. Eosinophils are thought to play a major role in AHR [28]. CF prevented the development of AHR (Figure 1A), lung inflammation (Figure 2), and airway eosinophilia (Figures 3 and 4) and decreased the level of Th2 cytokines (IL-5 and IL-13) (Figure 5A) in the BALF. These results show that CF inhibits lung allergic responses in the OVA-induced asthma model.

Asthma causes immune abnormalities in various cell populations. Thus, another goal of asthma research is to evaluate asthma-related changes in specific cell subpopulations. In the present study, immunophenotyping by flow cytometry showed a similar pattern as total lymphocyte numbers in the BALF and lungs. The effects of CF on leukocyte subsets in the lungs and BALF of asthmatic mice included changes in the number of Gr-1^+^/CD11b^+ ^granulocytes and CD3^-^/CCR3^+ ^eosinophils compared with the control group (Figure 4), and the decrease in CD3^-^/CCR3^+ ^eosinophils was accompanied by concurrent decreases in eosinophils in the BALF (Figure 2). CF also inhibited B-cell-dependent production of OVA-specific IgE in the serum (Figure 5C).

CC chemokine receptor 3 (CCR3) is expressed on eosinophils, mast cell (MC), basophils, and a subset of Th2 lymphocytes [29]. Eosinophils are attracted via CCR3 to chemoattractants such as eotaxin released in the airways of asthmatic patients [30]. The inhibition of pulmonary eosinophilia by CCR3 receptor blockade using certain antagonists may reduce inflammation and AHR in asthma. Moreover, eosinophils express Gr-1; therefore, eosinophil populations may constitute a substantial portion of the CD11b + Gr-1^+ ^cells. Our results (Figure 4) showed that the number of Gr-1^+ ^cells in the in BALF and lung increased with OVA challenge but significantly decreased with CF treatment.

We observed significant correlations between IL-5 levels and CCR3 expression on eosinophils. We hypothesised that CF prevents AHR by downregulating IL-5 production and thereby reducing eosinophilia.

Nuclear factor kappa B (NF-κB) is a major family of transcription factors that is activated during the inflammatory response in asthma. NF-κB is a key transcriptional regulator of multiple pro-inflammatory mediators such as TNFα and interleukins, and enhanced activation of NF-κB has been implicated in asthma [31].

The synthesis of many inflammatory mediators such as TNFα; IL-4; IL-5; IL-8; regulated upon activation, normal T-cell-expressed and secreted (RANTES); and eotaxins, which are thought to be important in asthma and COPD pathogenesis, is regulated through the activation of p38 mitogen-activated protein kinase (MAPK) [32]. p38 MAPK activates an inflammatory transcriptome similar to that of NF-κB and is activated in cells of patients with severe asthma [33]. An antisense oligonucleotide that blocks the expression of p38 MAPK showed inhibitory efficacy in a murine asthma model [34]. p38 MAPK plays a key role in the activation of GATA-binding protein 3 (GATA3), a transcription factor that regulates Th2 cell differentiation and the expression of Th2 cytokines [35]. The CF extract also suppressed an increase in the NF-κB levels [8]. An *in vivo *study showed that CF extract suppressed the acetic acid-induced writhing response in mice. An aqueous extract of CF exerts anti-inflammatory and analgesic effects by suppressing COX-2 and iNOS expression through decreased NF-κB binding [8].

According to a previous study [36], cornuside, a primary component of CF, decreases the LPS-stimulated phosphorylation of MAPKs (ERK1/2, p38, and JNK1/2) in a concentration-dependent manner. These results also indicate that among MAPK subtypes, JNK1/2 is inhibited most potently by cornuside. Many studies have suggested that MAPKs can participate in the regulation of NF-κB transcriptional activity. The activation of p38 but not ERK1/2 by LPS results in NF-κB activation and subsequent iNOS and NO release in macrophages. It is reasonable to assume that p38 suppression by cornuside inhibits the NF-κB pathway in LPS-treated RAW 264.7 cells [37].

The MUC5AC mucin protein is mainly expressed in goblet cells in the airway surface epithelium [38], and oleanolic acid and ursolic acid inhibit the production of MUC5AC induced by epidermal growth factor (EGF) and phorbol 12-myristate 13-acetate (PMA), respectively [39]. Although the underlying mechanisms of action of CF on airway inflammation and anti-asthmatic activity are not clear at present, from the aforementioned reports, it is likely that CF regulates the MAPK cascade via the EGF receptor and/or possible regulators of the NF-κB signalling pathway.

The inhibitory effects of CF on airway inflammation, hyperresponsiveness, and Th2 cytokine production might explain, at least in part, the traditional use of CF as an anti-inflammatory agent and potential anti-asthmatic therapy in Korean traditional medicine. It would be valuable to identify natural products that specifically inhibit airway inflammation, hyperresponsiveness, and Th2 cytokine production. Although the precise mechanism of action is not clear and requires further investigation, to our knowledge, this is the first study to show that CF significantly inhibits airway inflammation and hyperresponsiveness in a mouse model of allergic asthma. Therefore, we propose that CF be used for the treatment of pathologic inflammatory airway disorders such as allergic asthma.

## Conclusions

Our data indicate that CF has profound inhibitory effects on airway inflammation in a mouse model of asthma, and these effects were caused by the suppression of Th2 cytokines (IL-5), B cell-dependent production of OVA-specific IgE, and eosinophil CCR3 expression. It is reasonable to assume that the anti-inflammatory and anti-asthmatic activity of CF might be mediated by the inhibition of the NF-kB pathway. Hence, CF may act as a potential Th2 cytokine (IL-5) antagonist and have a therapeutic effect on allergic asthma.

## Abbreviations

AHR: airway hyperresponsiveness; Th: T helper cell; CF: Corni fructus; i.p.: Intraperitoneal; i.t.: Intratracheal; i.n.: Intranasal; OVA: Ovalbumin; IgE: Immuno-globulin E; IL: Interleukin; BALF: Bronchoalveolar lavage fluid; H&E: Haematoxylin-eosin; M-T: Masson-trichrome; PAS: Periodic acid-Schiff; ELISA: Enzyme-linked immunosorbent assay; HPLC: High performance liquid chromatography; ATP: Adenosine triphosphate; CCR3: CC chemokine receptor 3; MC: mast cell; NF-κB: Nuclear factor-kappaB; RANTES: Regulated on Activation: normal T cell-expressed and secreted; MAPK: Mitogen-activated protein kinase; GATA3: GATA-binding protein 3; EGF: Epidermal growth factor; PMA: Phorbol 12-myristate 13-acetate.

## Competing of interests

The authors declare that they have no competing interests.

## Authors' contributions

SH Kim, BK Kim, and YC Lee participated in the design of the study, data analyses, and manuscript preparation. SH Kim, BK Kim, and YC Lee conducted the assays and analyses. All authors read and approved the final manuscript.
